# Associations of Sedentary Time with Heart Rate and Heart Rate Variability in Adults: A Systematic Review and Meta-Analysis of Observational Studies

**DOI:** 10.3390/ijerph18168508

**Published:** 2021-08-12

**Authors:** Abdullah Bandar Alansare, Lauren C. Bates, Lee Stoner, Christopher E. Kline, Elizabeth Nagle, J. Richard Jennings, Erik D. Hanson, Mark A. Faghy, Bethany Barone Gibbs

**Affiliations:** 1 Department of Exercise Physiology, College of Sport Sciences and Physical Activity, King Saud University, King Khalid Rd, Riyadh 80200, Saudi Arabia; 2 Department of Exercise and Sport Science, University of North Carolina, Chapel Hill, NC 27599, USA; lbates15@live.unc.edu (L.C.B.); Stonerl@email.unc.edu (L.S.); edhanson@email.unc.edu (E.D.H.); 3 Department of Health and Human Development, School of Education, University of Pittsburgh, 140 Trees Hall, Pittsburgh, PA 15261, USA; chriskline@pitt.edu (C.E.K.); nagle@pitt.edu (E.N.); bbarone@pitt.edu (B.B.G.); 4 Department of Psychiatry, University of Pittsburgh, Pittsburgh, PA 15219, USA; jenningsjr@upmc.edu; 5 Human Sciences Research Centre, University of Derby, Derby DE22 1GB, UK; M.Faghy@Derby.ac.uk

**Keywords:** heart rate variability, sedentary time, lifestyle behaviors, autonomic regulation, vagal activity

## Abstract

Purpose: To evaluate if sedentary time (ST) is associated with heart rate (HR) and variability (HRV) in adults. Methods: We systematically searched PubMed and Google Scholar through June 2020. Inclusion criteria were observational design, humans, adults, English language, ST as the exposure, resting HR/HRV as the outcome, and (meta-analysis only) availability of the quantitative association with variability. After qualitative synthesis, meta-analysis used inverse variance heterogeneity models to estimate pooled associations. Results: Thirteen and eight articles met the criteria for the systematic review and meta-analysis, respectively. All studies were cross-sectional and few used gold standard ST or HRV assessment methodology. The qualitative synthesis suggested no associations between ST and HR/HRV. The meta-analysis found a significant association between ST and HR (β = 0.24 bpm per hour ST; CI: 0.10, 0.37) that was stronger in males (β = 0.36 bpm per hour ST; CI: 0.19, 0.53). Pooled associations between ST and HRV indices were non-significant (*p* > 0.05). Substantial heterogeneity was detected. Conclusions: The limited available evidence suggests an unfavorable but not clinically meaningful association between ST and HR, but no association with HRV. Future longitudinal studies assessing ST with thigh-based monitoring and HRV with electrocardiogram are needed.

## 1. Introduction

Cardiovascular disease (CVD) remains the leading cause of death in adults worldwide [1,2]. Recent evidence indicates that sedentary behavior (i.e., any waking behavior that has an energy expenditure of ≤1.5 metabolic equivalents and occurs in lying, reclining, or seated position [3]) is associated with CVD incidence and mortality [4,5]. Importantly, this association is distinct from the harmful impacts of physical inactivity, which is defined as not engaging in sufficient levels of moderate-to-vigorous intensity physical activity (MVPA) [6]. This association was graded as ‘strong’ by the 2018 Physical Activity Guidelines Advisory Committee [7]. However, the mechanisms by which greater sedentary time (ST) leads to elevated CVD risk remain unclear. 

Cardiac-autonomic dysregulation is a physiological mechanism that links risk factors such as hypertension and diabetes to CVD outcomes [8]. Cardiac-autonomic regulation is commonly evaluated by measuring resting heart rate [9,10] and/or heart rate variability (HRV), which is the variation in time intervals between consecutive heartbeats [11,12,13]. HRV is commonly operationalized into time and frequency domain indices [13]. Some, but not all, of these HRV indices have a well-understood physiological and statistical basis [13]. For example, the standard deviation of normal R-R intervals (SDNN) is a time domain index that represents the overall variability due to cardiac sympathetic and parasympathetic activity [13]. Moreover, the root mean square of the successive differences (RMSSD; a time domain index) and high frequency (HF; a frequency domain index) are measures of cardiac parasympathetic activity, which is modulated by respiration [14]. On the other hand, low frequency (LF) is a frequency domain index that has been used to represent resting cardiac-sympathetic activity; however, recent evidence indicates that LF is not a measure of resting cardiac-sympathetic activity [15,16]. Regardless of their physiological meanings, consistent evidence has specified that higher resting HR and lower HRV are indicative of increased cardiac sympathetic activity and/or decreased cardiac parasympathetic activity and suggests vulnerability to CVD [9,10,17].

Recently, higher resting HR and lower HRV have been proposed as an important linking mechanism between ST and CVD [18,19,20]. However, observational studies evaluating the association between ST and HR or HRV in adults have reported inconsistent results including negative, positive, and null associations [18,20,21,22,23,24,25,26]. To address this uncertainty, we systematically reviewed the current evidence relating ST to HR and HRV to shed light on whether this proposed mechanism is supported by the available research and to clarify the potential role of cardiac-autonomic dysfunction in the association of ST with CVD and mortality. Thus, the primary aim of this study was to qualitatively summarize and quantitatively synthesize the available evidence from observational studies examining the association of ST with HR and HRV in adults. It was hypothesized that higher time spent in sedentary behavior would be associated with higher resting HR and lower resting overall variability, indicating cardiac-autonomic dysregulation in adults.

## 2. Materials and Methods

This systematic review and meta-analysis was registered in the International Prospective Register of Systematic Reviews (PROSPERO) database (registration ID: CRD42020196516) and was performed according to Preferred Reporting Items for Systematic Review and Meta-Analysis (PRISMA) guidelines [27].

### 2.1. Data Sources and Search Strategy

PubMed and Google Scholar were searched systematically by two independent researchers (AA and LB) using the following terms: (“sitting” OR “sedentarism” OR “sedentary” OR “television time” OR “screen time”) AND (“HRV” OR “heart rate variability” OR “heart rate”). Reference lists of all identified trials and relevant reviews or editorials were also manually examined. The search was limited to include only published studies between database inception and 3 June 2020. 

### 2.2. Study Selection

Two independent reviewers (ABA and LCB) comprehensively screened the titles, abstracts, and entire manuscripts, when needed, of all the identified studies using the inclusion criteria listed below. Any discrepancies for inclusion between reviewers were settled by consensus or, when necessary, a third reviewer (BBG or LS). Inclusion criteria for the systematic review were: (1) human participants ≥18 years old; (2) English language; (3) observational research designs including cross-sectional, longitudinal, or case-control studies; (4) objective or self-reported measures of ST including total, bouts, and domain-specific ST as the exposure variable; and (5) at least one reported outcome of interest including resting HR and/or HRV indices (e.g., the standard deviation of normal R-R intervals [SDNN], root mean square of successive differences [RMSSD], low frequency [LF], higher frequency [HF], and LF/HF ratio). The same inclusion criteria were used for the meta-analysis with the addition of a reported estimate of the association between ST and HR or HRV. For HR, we required the difference in outcome per unit increase in exposure (β) or a correlation coefficient (*r*) along with some measure of variability that would allow β calculation (e.g., standard deviation). For HRV, we required a correlation coefficient (*r*) or a regression coefficient (β) along with some measure of variability that would allow for *r* calculation. 

### 2.3. Data Extraction and Quality Assessment

Two independent reviewers (ABA and BBG) extracted data from each eligible article including the name of the first author and year of publication, country, characteristics of the sample, method of assessment for ST, method of assessment of HR and/or HRV, and number and description of estimates. To calculate the pooled effect, associations between ST and HR and/or HRV indices were extracted or calculated using reported β, *r*, standard deviations, and sample sizes (manual calculations are described below). An eligible article could have reported more than one estimate across subgroups [18,21,25] or within subjects (i.e., separately for occupational and leisure-time sedentary behavior, or separately for weekend and weekday ST) [20,21,22]; in such cases, data for each estimate were extracted separately. If articles had a missing estimate and lacked sufficient data for manual calculation, corresponding authors were contacted. If authors failed to provide necessary data, their study was retained in the systematic review but excluded from the meta-analysis. 

Two authors (ABA and BBG) adapted the NIH Quality Assessment Tool for Observational Cohort and Cross-Sectional Studies quality score [28] to measure 13 specific quality elements (e.g., objective of the study, risk of selection and measurement biases, evaluation of temporality, validity and reliability of measurement methodology) important for rigor when evaluating associations between ST and HR or HRV. Each element was assigned one point if the answer was yes and zero points if the answer was no. Total points of all elements were aggregated to calculate the final quality score for each article, which could range from 0 to 13 points.

### 2.4. Data Synthesis

All studies meeting the inclusion criteria were qualitatively (descriptive) and semi-quantitatively synthesized. Semi-quantitative synthesis counted the number of articles with direct, null, or inverse associations and arrived at an overall interpretation using previously published methods [29]: (1) “no association” if >50% of the studies reported null findings; (2) “inconclusive” if exactly 50% of the studies reported no associations and 50% of the studies demonstrated significant in one direction (positive or negative) association; (3) “some evidence for association” if >50% of the studies demonstrated a positive (or negative) association; and (4) “consistent evidence for association” if all the studies (100%) showed significant association in a positive (or negative) direction. 

For the quantitative synthesis (meta-analysis), we applied a correction by adjusting the sample size (adjusted *n* = original *n*/number of estimates) for any estimate from an article reporting more than one estimate either across subgroups or within-subjects. This allowed for the use of all estimates in the pooled effect size calculation by correcting (reducing) weights of multiple estimates from the same study [30]. For associations between ST and HR, the pooled effect size (β) was calculated as the change in HR (bpm) per unit increase of ST (hour/day) with a 95% confidence interval. Estimates not reported per hour of ST were scaled appropriately. Studies reporting Pearson’s *r* rather than β were converted using the following formula: β = *r* * (standard deviation of the outcome/standard deviation of exposure). Standard error (SE) was calculated as √[(1−*r*^2^)/(n−2)] or estimated from 95% confidence intervals if not specifically reported [31]. HRV indices were reported using various units and some studies reported more than one measurement unit simultaneously. Thus, we systematically extracted HRV indices according to the following priority order regarding measurement unit: (1) natural log of milliseconds/milliseconds squared; (2) milliseconds/milliseconds squared; (3) percentage; and (4) normalized units. Due to this heterogeneity in the measurement units, associations between ST and HRV were extracted as or converted to unitless correlation coefficients (*r*) and then combined in a pooled estimate [32]. If not reported, Pearson’s r was manually calculated as follows: *r* = standardized β + 0.05λ, where λ = 1 if the standardized β is positive and λ = 0 if the standardized β is negative [33]. 

### 2.5. Data Analysis

The Stata Metan package (Stata Statistical Software; StataCorp, LLC, College Station, TX, USA) and MetaXL software (https://www.epigear.com/index_files/metaxl.html, accessed on 26 June 2019) were utilized to perform the meta-analyses. If there were three or more estimates with the same outcome measure, a meta-analysis was conducted. Because the included studies had substantial between-article variability in the sample characteristics, measurement methodology for ST, HR, and HRV assessment, the inverse variance heterogeneity (IVHET) model was selected to account for the potential heterogeneity [34]. The association between ST and HR was pooled and reported as β (bpm per hour of ST). Furthermore, Cohen’s *d* was calculated as the pooled β (bpm per hour ST)/median standard deviation of baseline HR across studies (bpm). The magnitude of association was evaluated using Cohen’s *d* as follows: *d* < 0.2 is trivial; *d* = 0.2 is small; *d* = 0.5 is moderate; and *d* = 0.8 is large [35]. On the other hand, the association between ST and HRV was pooled and reported as *r*, which was also used to evaluate the magnitude of the association as follows: *r* = 0.2 is small; *r* = 0.5 is medium; *r* = 0.8 is large [36]. 

Subsequent to running the IVHET models, we examined the robustness of the pooled results and the potential for publication bias. Sensitivity analyses removed one study at a time to test the robustness of the pooled results. If the pooled estimate was altered in statistical significance or magnitude of effect grading by removing any one study, the pooled estimate was reported with and without that study. Though we were unable to visually evaluate publication bias by the Begg’s funnel plot test due to less than 10 included studies [37], we statistically evaluated publication bias by Egger’s regression test [38]. Finally, statistical heterogeneity was assessed by the I^2^ statistic, where <25% indicates low risk of heterogeneity, 25–75% indicates a moderate risk of heterogeneity, and >75% indicates a considerable risk of heterogeneity [39]. If sufficient data were available (at least three unique studies across at least two sub-groups), the following prespecified sub-group analyses were conducted to explore potential sources of heterogeneity: sex (i.e., male, female, or combined), sedentary behavior assessment (i.e., subjective vs. objective), sedentary behavior domain (i.e., total, television only, leisure, occupational), timing of HR or HRV measurement (i.e., nocturnal vs. diurnal), using ECG to measure HRV (yes vs. no), and covariate adjustment for MVPA (yes vs. no).

## 3. Results

### 3.1. Literature Search and Trial Selection

**Figure 1:**
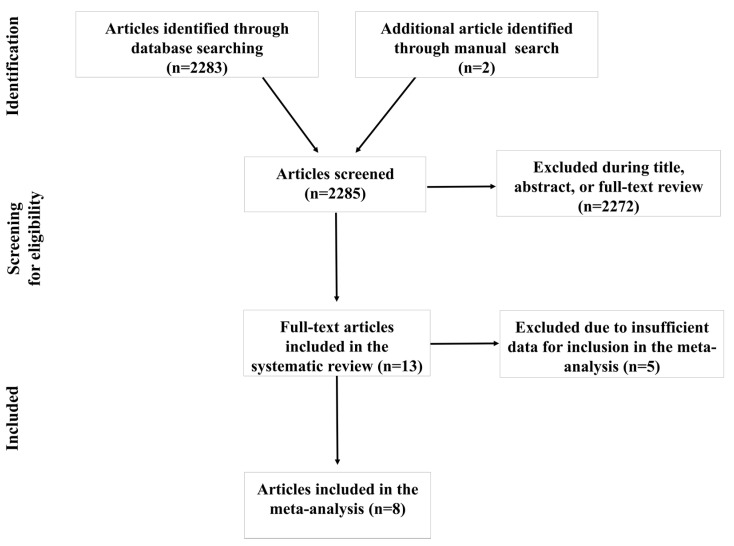
Article selection flow chart.

Figure 1 displays the results of the systematic literature search. Using the predetermined terms and filters, 2283 articles were initially found through database searching. Two additional articles were manually identified. Following a comprehensive examination of titles, abstracts, and full text when needed, 2272 articles were excluded due to not meeting one or more inclusion criteria. Thirteen articles met the inclusion criteria for the systematic review. Though only eight articles met the criteria for inclusion in the meta-analysis, these articles yielded a total of 19 estimates. The remaining five articles did not report estimates of associations and the corresponding authors did not provide the necessary quantitative data to calculate the pooled effects.

### 3.2. Characteristics of Included Articles and Quality Assessment 

The characteristics of the included articles in the systematic review and meta-analysis are presented in Table 1. The included articles were published between 2011 and 2020. The populations were from Australia [21], Brazil [23,26,40,41,42], Canada [43], Denmark [20,22], Finland [25], Spain [44], the United Kingdom [24], and Sweden [18]. The sample sizes of the included articles ranged from 35 [26] to 46,832 [18]. One article included only male participants [26], and the remaining included both sexes and analyzed them either together [20,22,23,24,40,41,42,43,44] or separately [18,21,25]. To measure ST, five articles utilized self-report instruments [18,21,26,40,44] and eight used objective devices [20,22,23,24,25,41,42,43]. Furthermore, the included studies in the meta-analysis reported either a single estimate [23,24,26], multiple within-subject estimates [20,21,22], or multiple subgroup estimates [18,21,25]. Finally, out of the thirteen included articles in the systematic review, one had a quality score of nine [20], one had a quality score of eight [22], four had a quality score of seven [18,25,41,43], three had a quality score of six [21,26,44], three had a quality score of five [24,41,42], and one had a quality score of four [23]. In general, most studies earned quality points for stating a research objective, evaluating ST as a continuous outcome, and for a low chance of selection bias. Studies typically had lower scores due to cross-sectional designs, failure to use a thigh-based accelerometer to measure ST, and failure to measure ST at more than one timepoint (Supplementary Table S1).

Regarding outcomes (Table 2), five articles measured only HR [18,21,40,43,44], six measured only HRV [20,23,24,26,41,42], and two measured both HR and HRV [22,25]. HR and/or HRV were measured using ECG [20,22,24], HR monitors [23,25,26,41,42], or oscillometers (HR only) [18,40,43]; two studies did not report the device used [21,44]. When reported and not including oscillometer HR measurements, the duration of the HR and HRV measurement ranged from three minutes [25] to three x five-minute segments [20,22] and were performed during the daytime [18,21,23,24,25,40,41,42,43,44], in the afternoon [26], or at night [20,22]. The measurements of HR and HRV were obtained in supine [20,22,23,26,41,42] and seated postures [18,21,25,40,43], with posture not reported in two articles [24,44].

### 3.3. Association between Sedentary Time and Heart Rate

For the qualitative assessment, 53% (*n* = 17) of estimates detected no relationship between ST and HR. Because more than 50% of estimates reported null findings, the qualitative synthesis suggested no association between ST and HR. 

**Figure 2:**
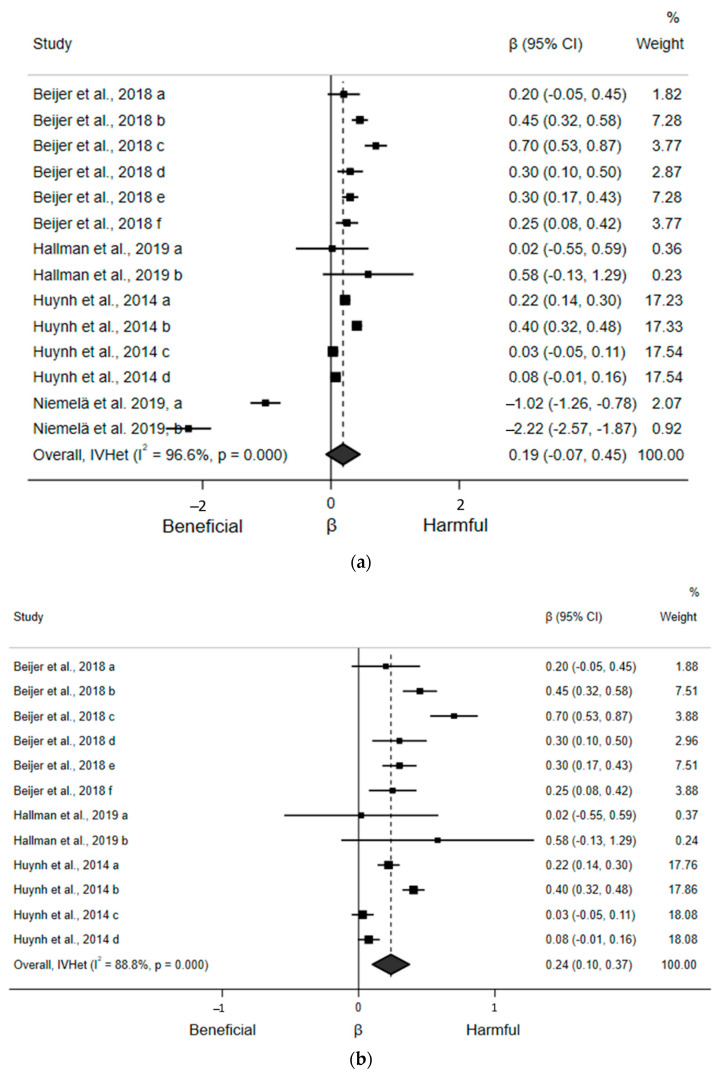
Forest plots of articles examining the association between ST and HR. (**a**) Association between ST (hours/day) and HR (beats/minute) including all articles. (**b**) Association between ST (hours/day) and HR (beats/minute) after excluding the high influence article [25].

For the quantitative evaluation, 14 estimates from the four included articles found a trivial and statistically nonsignificant association between ST and HR, where each hour increase in ST was associated with a 0.19 bpm (95% CI: −0.07, 0.45; *d* = 0.03) increase in HR (Figure 2a). However, sensitivity analyses removing one article at a time suggested one article substantially altered the statistical inference of the pooled estimate [25]. The direction of the association in this article opposed the direction of the association in all other articles; when reanalyzed after excluding the influential article, the association became statistically significant but remained trivial, with each hour increase in ST associated with a 0.24 bpm (95% CI: 0.10, 0.37; *d* = 0.04) increase in HR (Figure 2b). Egger’s regression test indicated no asymmetry (β_0_ = −1.29; *p* = 0.57). There was statistically significant and considerable heterogeneity (I^2^ = 96.6%, *p* < 0.001). Due to an insufficient number of estimates within subgroups, we were only able to perform a subgroup analysis by sex, which found a significant association between ST and HR in males but not in females or mixed-sex populations (Supplementary Figure S1).

### 3.4. Association between Sedentary Time and Time Domain Indices of Heart Rate Variability

For the qualitative assessment, 80% (*n* = 5) and 71% (*n* = 7) of the estimates found no correlation between ST and SDNN and RMSSD, respectively. Because more than 50% of the estimates reported null findings, the qualitative synthesis suggested no association between ST and SDNN or RMSSD. 

**Figure 3:**
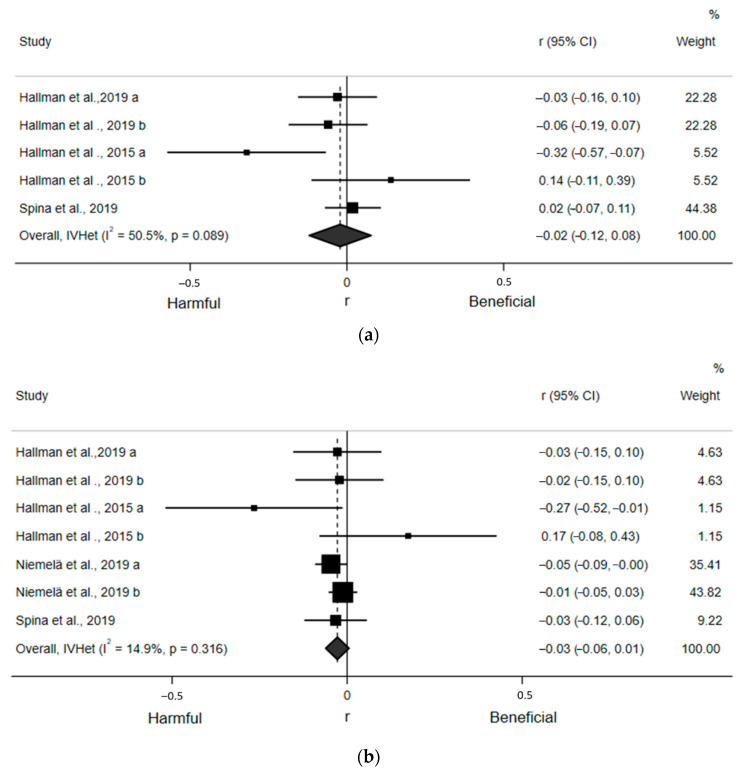
Forest plots of articles examining the association between ST and time domain indices of HRV. (**a**) Association between ST and SDNN. (**b**) Association between ST and RMSSD.

For the quantitative assessment, five estimates from the three included articles found a statistically nonsignificant, small, and inverse correlation (r = −0.02; 95% CI: −0.12, 0.08) between ST and SDNN (Figure 3a). Egger’s regression test indicated no asymmetry (β_0_ = −1.34; *p* = 0.27). There was a moderate, but not statistically significant, risk of heterogeneity (I^2^ = 50.5%, *p* = 0.089). Similarly, seven estimates from the four included articles found a non-statistically significant, small, and inverse correlation (r = −0.03; 95% CI: −0.06, 0.01) between ST and RMSSD (Figure 3b). Egger’s regression test (β_0_ = −0.15; *p* = 0.83) indicated no asymmetry. There was also low and statistically nonsignificant heterogeneity (I^2^ = 14.9%, *p* = 0.32). Sensitivity analyses removing one article at a time suggested no article statistically and significantly influenced these pooled estimates. None of our prespecified subgroup analyses could be conducted due to an insufficient number of estimates within subgroups.

### 3.5. Association between Sedentary Time and Frequency Domain Indices of Heart Rate Variability

For the qualitative assessment, 87% (*n* = 8), 100% (*n* = 6), and 90% (*n* = 10) of the estimates detected no correlation between ST and LF, HF, and LF/HF ratio, respectively. Because more than 50% of the estimates reported null findings, the qualitative synthesis indicated no association between ST and LF, HF, or LF/HF ratio.

**Figure 4:**
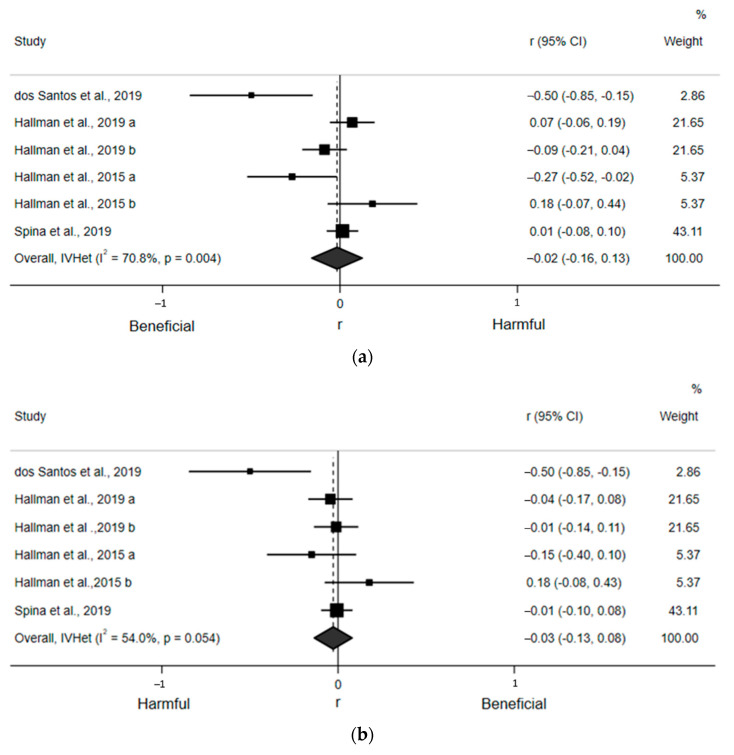

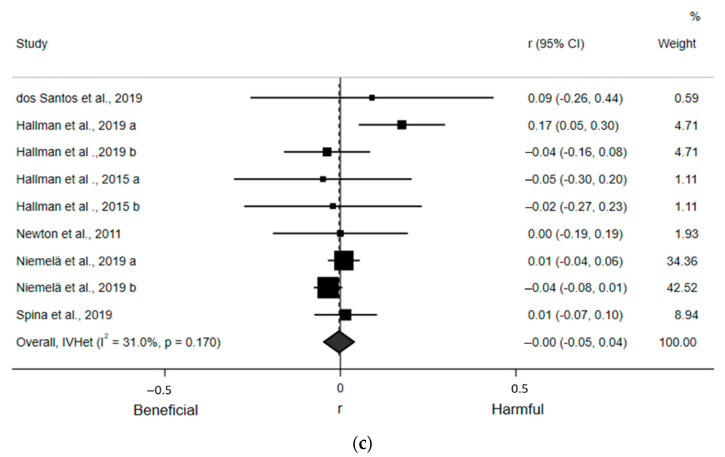
Forest plots of articles examining the association between ST and frequency domain indices of HRV. (**a**) Association between ST and LF. (**b**) Association between ST and HF. (**c**) Association between ST and LF/HF ratio.

For the qualitative assessment, six estimates from the four included articles found a statistically nonsignificant, small, and inverse correlation (r = −0.02; 95% CI: −0.16, 0.13) between ST and LF (Figure 4a). Egger’s regression test indicated no asymmetry (β_0_ = −2.60; *p* = 0.12). There was also moderate and statistically significant heterogeneity (I^2^ = 70.8%, *p* = 0.004). Likewise, six estimates from the four included articles detected a statistically nonsignificant, small, and inverse correlation (r = −0.03; 95% CI: −0.13, 0.08) between ST and HF (Figure 4b). Egger’s regression test indicated no asymmetry (β_0_ = −1.46; *p* = 0.13). There was moderate, but not statistically significant, heterogeneity (I^2^ = 54.0%, *p* = 0.054). Finally, nine estimates from the six included articles revealed a statistically nonsignificant and negligible correlation (r = −0.00; 95% CI: −0.05, 0.04) between ST and the LF/HF ratio (Figure 4c). Egger’s regression test (β_0_ = 0.37; *p* = 0.59) indicated no asymmetry. There was moderate, but not statistically significant, heterogeneity (I^2^ = 31.0%, *p* = 0.17). Sensitivity analyses removing one article at a time suggested no article statistically and significantly affected any of these pooled estimates. Due to an insufficient number of estimates within subgroups, we were only able to perform subgroup analyses for LF/HF ratio by adjustment for MVPA and using ECG, which yielded small and statistically nonsignificant associations (Supplementary Figure S2a,b).

## 4. Discussion

This study was the first systematic review and meta-analysis to synthesize the existing literature on the observational association of ST with HR and HRV in adults. Only thirteen studies have assessed the association of ST with HR and HRV. Among these studies, study quality was generally low with studies, on average, only meeting six of 13 quality criteria. Overall, we found a statistically significant and direct, yet trivial, association between ST and HR. Subgroup analysis found this association to be only apparent in males. However, there were no associations between ST and any of the HRV indices. 

### 4.1. Association between Sedentary Time and Heart Rate

Though our meta-analysis statistically supported our hypothesis that higher ST would be associated with greater HR, this association was very small. Each hour increase in ST was associated with a 0.24 bpm increase in HR, corresponding to *d* = 0.04, which is likely to be of minimal clinical significance. This small effect can be benchmarked against a previous meta-analysis reporting that a 10-bpm increase in resting HR was associated with 9% and 8% higher risk of all-cause and cardiovascular mortality, respectively [45]. Thus, greater HR is unlikely to explain much of the association between ST and CVD and mortality. Conceivably, other cardiovascular mechanisms such as impaired metabolic and vascular function may explain the association between higher ST and CVD and mortality [46]. 

Of note, we detected considerable risk of heterogeneity in the pooled HR estimate. Our sex subgroup analysis revealed that the association of ST with HR was stronger in males (Supplementary Figure S2). This disparity may be partially explained by sex differences in the autonomic control of the heart, where females usually have higher vagal activity and lower sympathetic activity, granting them cardioprotective effects compared to males [47]. As such, the observed impact of sedentary behavior on HR might be attenuated in females compared to males. Other potential factors (e.g., ST measurement instruments, domain of ST, posture of HR measurement) may explain more of this considerable heterogeneity in the association between ST and HR and warrant further investigation. For example, two of the four included HR studies in the meta-analysis used self-report instruments [18,21], which are subject to measurement error that could have affected the association between ST and HR [48]. Additionally, two of the four studies examined the association between ST and HR using domain-specific components of ST [18,22]. Though not fully understood yet, the association between ST and HR may differ by sedentary behavior domains as it does with other health outcomes [49,50]. Finally, methodological issues such as whether pre-visit abstention from PA and food/caffeine/nicotine intake were implemented could have contributed to the observed heterogeneity [51,52]. These factors were not frequently mentioned or accounted for in the articles included in this review; future research should account for these factors when assessing HR. 

### 4.2. Association between Sedentary Time and Heart Rate Variability

It was hypothesized that higher ST would be associated with lower overall (i.e., lower SDNN) and cardiac-parasympathetic HRV indices (i.e., lower RMSSD and HF). However, our meta-analyses revealed no associations between ST and HRV indices (i.e., SDNN, RMSSD, LF, HF, LF/HF ratio). These conclusions were limited due to the low quality and small number of published studies; this also prevented in-depth examinations using subgroup analyses. Still, these findings suggest that higher ST may not be associated with cardiac-autonomic impairment. However, an important consideration when interpreting these findings is that HRV is only able to measure overall and cardiac-parasympathetic activity when resting. Although LF used to be thought of as a measure of cardiac-sympathetic activity, recent studies have found that LF does not actually correspond to resting cardiac-sympathetic activity [15,16] for the following reasons. First, studies have shown that LF fails to correlate to the “gold standard” measure of cardiac-sympathetic activity (i.e., cardiac norepinephrine spillover) at rest. Additionally, cardiac-sympathetic blockade by segmental spinal anesthesia has no impact on LF. Finally, the administration of a sympathetic agonist, which increases both heart rate and norepinephrine level, appears to have no influence on LF [15]. Thus, though LF (and therefore HRV) was formerly thought to measure both resting cardia-sympathetic and cardiac-parasympathetic regulation, the current understanding is that HRV is limited to the measurement of total variability and cardiac-parasympathetic activity [13,14]. Therefore, these findings do not provide data to evaluate whether higher ST is associated with cardiac-sympathetic overactivation, a mechanism that has been specifically hypothesized as the primary autonomic pathway linking ST with CVD [46]. Future studies evaluating ST with specific cardiac-sympathetic overactivation as well as addressing the noted limitations of the current research are needed to better understand whether autonomic dysfunction is indeed a mechanism linking ST and CVD. 

Similar to our pooled HR estimate, there were several potential sources of heterogeneity (e.g., participant characteristics, ST measurement instruments, domain of ST, duration and posture of HRV measurements, and HRV measurement devices) that could have impacted our pooled HRV estimates. However, we could only perform limited subgroup analyses due to the insufficient number of estimates within subgroups. The few subgroup analyses we were able to conduct did not identify factors likely to be responsible for the observed heterogeneity. Furthermore, our pooled null estimates were potentially affected by several important methodological aspects of the available studies including cross-sectional designs and the lack of control for important covariates such as respiration rate, which can significantly affect HRV [53]. Altogether, though our pooled HRV estimates found no relationship, the observed considerable heterogeneity indicates that there may be more than one true underlying association between sedentary behavior and HRV.

### 4.3. Hypothesized Physiological Mechanisms

It has been proposed that frequent exposure to adverse, acute cardiovascular responses to sedentary behavior could manifest as chronic associations between high levels of sedentary behavior with increased HR and decreased HRV. Acutely, sedentary behavior such as prolonged sitting causes blood pooling in the lower extremities leading to interrupted blood flow and reduced blood pressure [54]. To compensate, the sympathetic nervous system would increase its outflow and the vagus nerve would likely reduce its outflow to increase HR to adjust blood flow and pressure [54,55,56]. This would lead to reduced overall and cardiac-parasympathetic HRV indices [57,58]. Concurrently, this decrease in blood flow causes a reduction in shear stress and, eventually, nitric oxide (NO) bioavailability [54,59]; additionally, with regard to its vasodilatory effect, NO acts as a vagal activity enhancer, leading to augmented acetylcholine release [60]. Thus, reduction in NO bioavailability may attenuate acetylcholine release and, therefore, HRV. Repeated exposure to sedentary behavior and these resulting responses were hypothesized to manifest as chronic augmentation in HR and reduction in HRV. However, our findings do not lend support to a theory where repeated exposure to these acute physiological responses would lead to chronic, adverse effects. 

### 4.4. Limitations

Several limitations should be considered when interpreting our results. First, the currently published observational studies relating ST to HR and HRV all had cross-sectional designs, which are susceptible to biases such as residual confounding and reverse causality. In addition, significant heterogeneity was observed. This may be partly explained by differences across ST measurement instruments and domains. For example, only two studies used a thigh-worn accelerometer [20,22], which is the gold standard measure of ST because it can accurately distinguish between seated and standing postures and can therefore provide a more precise ST estimation compared to wrist- or waist-worn monitors or self-reporting tools [48,61,62]. As such, our pooled estimates were likely impacted due to ST measurement error in the remaining studies [23,24,25,26]. Furthermore, growing evidence indicates that various domains of ST relate differently to a variety of health outcomes [49,50]. Our pooled estimates could not account for such potential disparities due to the insufficient number of studies within subgroups. 

Equally important, there are other limitations related specifically to HRV that potentially influenced our pooled estimates. Because HRV is a time-dependent measure [63] and short- vs. long-term HRV is affected by different mechanisms [64], it may be inappropriate to compare and aggregate HRV indices that were obtained from different measurement durations [63]. Moreover, another notable limitation is that only one study [23] accounted for respiration rate, which could have significantly affected our pooled estimates [53,65]. Furthermore, HRV measurement posture (i.e., seated, supine, standing) and timing (i.e., morning, afternoon, nocturnal) varied substantially. Both of these measurement-related factors could have introduced variability and influenced our pooled estimates [66]. Finally, only half of the studies [23,25,26] assessed HRV with the gold standard technique of ECG [63]; this represents another important limitation that might have influenced our pooled estimates. This was suggested when we compared the LF/HF ratio estimates based upon whether ECG was used; the pooled associations were in the opposing direction, though both were statically nonsignificant (Supplementary Figure S2b). Unfortunately, due to the limited number of available studies, we were unable to perform this subgroup analysis for other HRV indices. 

### 4.5. Implications and Future Directions 

The findings of this systematic review and meta-analysis do not support the hypothesis that HR and HRV are mechanisms linking sedentary behavior and CVD. As the association between sedentary behavior and CVD is more established [46], our systematic review and meta-analysis suggest that there might be other more important mechanisms (e.g., vascular dysfunction, metabolic disturbances, or sympathetic overactivation) that might explain such associations. However, given the above-mentioned limitations to the available data, future research should consider and address existing methodological limitations to confirm our null findings with greater rigor. Specifically, there is a need for studies that have longitudinal designs to establish temporality in the association between ST and HR and HRV. In addition, intervention studies that examine the effects of reducing ST on HR and HRV may be the best design to assess causality between ST and cardiac autonomic regulation. Studies should also utilize the gold standard techniques to measure ST (i.e., a thigh-worn monitor) and HRV (i.e., ECG with guideline-based processing and accounting for respiration rate). Other sources of potential heterogeneity that we were unable to disentangle may also be important future research directions such as preexisting CVD, domains and patterns of ST, and diurnal vs. nocturnal HRV measurement [67,68,69,70]. Finally, given that the COVID-19 pandemic has negatively affected lifestyle behaviors and mental health [71], future studies should investigate the role of the pandemic in the associations between ST and HRV.

## 5. Conclusions

Overall, the available, low-quality evidence suggests an unfavorable but not clinically meaningful association between ST and HR, but no association between ST and HRV. These results do not support the hypothesis that increases in HR and decreases in HRV are mechanisms linking increased ST and CVD. Future longitudinal research using optimal and standardized measurement methodology for sedentary behavior and HRV as well as the evaluation of potential sources of heterogeneity is needed to draw more comprehensive conclusions.

## Figures and Tables

**Table 1:** Population characteristics, ST measurement, subgroups, and quality of included articles.

Reference	Country	Sample N (% Male); Mean Age	ST Measurement	Description of Estimates	Quality
Included in meta-analysis and systematic review					
Beijer et al., 2018 [18]	Sweden	46,832 (41%); 47 yr	self-reported TV watching	age x sex subgroup estimates a (M; 26 y) b (M; 47 y) c (M; 68 y) d (F; 26 y) e (F; 47 y) f (F; 68 y)	7
dos Santos et al., 2019 [26]	Brazil	35 (100%); NR	self-reported sitting time	single estimate	6
Hallman et al., 2019 [22]	Denmark	490 (56%); 45 yr	thigh + trunk-mounted accelerometer	domain-specific estimates a (occupational sitting time) b (leisure sitting time)	8
Hallman et al., 2015 [20]	Denmark	126 (55%); 46 yr	thigh + trunk-mounted accelerometer	domain-specific estimates a (occupational sitting time) b (leisure sitting time)	9
Huynh et al., 2014 [21]	Australia	2328 (49%); 31 yr	self-reported sitting time	type of day × sex estimates a (M; weekday sitting time) b (M; weekend sitting time) c (F; weekday sitting time) d (F; weekend sitting time)	6
Newton et al., 2011 [24]	United Kingdom	107 (NR); NR	multi-sensor armband	single estimate	5
Niemelä et al., 2019 [25]	Finland	4150 (45%); 47 yr	wrist-worn accelerometer	sex subgroup estimates a (M) b (F)	7
Spina et al., 2019 [23]	Brazil	485 (37%); 48 yr	waist-worn accelerometer	single estimate	4
Included in systematic review only					
Delfino et al., 2020 [40]	Brazil	245 (24%); 45 yr	self-reported sitting time		7
Gerage et al., 2015 [41]	Brazil	87 (21%); 58 yr	hip-worn accelerometer		5
McGregor et al., 2018 [43]	Canada	7776 (50%); 47 yr	hip-worn accelerometer		7
Oliveira et al., 2020 [42]	Brazil	64 (14%); 39 yr	wrist-worn accelerometer		5
Recio-Rodriguez et al., 2013 [44]	Spain	732 (41%); 57 yr	self-reported TV watching		6

N: sample size; N/A: not applicable; NR: not reported; ST: sedentary time; yr: years.

**Table 2:** Description of outcomes from the included articles.

Reference	Outcome(s)	HR or HRV Measurement Device	HR or HRV Measurement Duration, Posture, and Type
Included in meta-analysis and systematic review			
Beijer et al., 2018 [18]	HR	oscillometer	N/A; seated; resting daytime
dos Santos et al., 2019 [26]	HRV (HF, LF, LF/HF)	HR monitor	5 min; supine; afternoon
Hallman et al., 2019 [22]	HR; HRV (SDNN, RMSSD, HF, LF, LF/HF)	ECG	3 × 5-min; supine; nocturnal
Hallman et al., 2015 [20]	HRV (SDNN, RMSSD, HF, LF, LF/HF)	ECG	3 × 5-min; supine; nocturnal
Huynh et al., 2014 [21]	HR	NR	NR; seated; resting daytime
Newton et al., 2011 [24]	HRV (LF/HF)	ECG	10 min; NR; daytime
Niemelä et al., 2019 [25]	HR; HRV (RMSSD, LF/HF)	HR monitor	5 min; seated and standing; daytime
Spina et al., 2019 [23]	HRV (SDNN, RMSSD, HF, LF, LF/HF)	HR monitor	5 min; supine; resting daytime
Included in systematic review only			
Delfino et al., 2020 [40]	HR	oscillometer	N/A; seated; resting daytime
Gerage et al., 2015 [41]	HRV (HF, LF)	HR monitor	5 min; supine; resting daytime
McGregor et al., 2018 [43]	HR	oscillometer	N/A; seated; resting daytime
Oliveira et al., 2020 [42]	HRV (LF, HF, LF/HF)	HR monitor	10 min; supine; resting daytime
Recio-Rodriguez et al., 2013 [44]	HR	NR	NR; NR; daytime

HR; heart rate, HRV; heart rate variability, N/A; not applicable, NR; not reported, SDNN; standard deviation of normal R-R intervals, RMSSD; root mean square of successive differences, LF; low frequency, HF; high frequency.

## Data Availability

The raw data supporting our conclusions will be made available by the corresponding author upon request.

## Supplementary Materials

The following are available online at https://www.mdpi.com/article/10.3390/ijerph18168508/s1, Table S1. Quality assessment of the included studies, Figure S1: Forest plots of association between ST and HR in subgroup analyses by sex, Figure S2: Forest plots of the association between ST and LF/HF ratio in subgroup analyses (a) Subgroup analyses by MVPA adjustment and (b) subgroup analysis by using electrocardiogram (ECG).
