# Supplementary material for: Associations of Sedentary Time with Heart Rate and Heart Rate Variability in Adults: A Systematic Review and Meta-Analysis of Observational Studies
Source: Int J Environ Res Public Health. 2021 Aug 12;18(16):8508. doi: 10.3390/ijerph18168508 (PMC8391190; doi:10.3390/ijerph18168508)
Supplement: Supplementary file 1 [file ijerph-18-08508-s001.zip › ijerph-1304831-supplementary.pdf]

Table S1. Quality Assessment of the Included Studies

Reference	Was the research question or objective in this paper clearly stated?	Was the study population clearly specified (i.e., demographics, period, location)?	Was the sample population-based?	Were all the subjects selected or recruited from the same or similar populations (including time period)? Were inclusion and exclusion criteria for being in the study prespecified and applied uniformly to all participants?	Was a sample size justification, power description, or variance and effect estimates provided?	For the analyses in this paper, Was temporality established?	Was the timeframe sufficient so that one could reasonably expect to see an association between ST and HR/HRV if it existed?	For ST, did the study examine different levels? (e.g., categories or continuous)?	Was ST assessed using validated, objective, thigh-worn device?	Was ST assessed more than once over time?	Was HR/HRV measured with valid and reliable methods, including pre-visit abstention from physical activity and intake (dietary, caffeine, nicotine) if appropriate?	Was HR objectively measured and HRV was measured by ECG (gold standard), if applicable?	Were key potential confounding variables measured and adjusted statistically for their impact on the relationship between ST and HR/HRV (specifically, age, sex, MVPA)?	Quality score (out of 13)
**Beijer et al., 2018 [12]**	■	■	■	■				■				■	■	**7**
**dos Santos et al., 2019 [20]**	■			■	■	■		■			■			**6**
**Hallman et al., 2019 [16]**	■	■		■				■	■		■	■	■	**8**
**Hallman et al., 2015 [14]**	■	■		■	■			■	■		■	■	■	**9**
**Huynh et al., 2014 [15]**	■	■	■	■				■					■	**6**
**Newton et al., 2011 [18]**	■	■		■				■				■		**5**
**Niemelä et al., 2019 [19]**	■	■	■	■				■			■		■	**7**

**Spina et al., 2019 [17]**	■			■		■	■			**4**
**Delfino et al., 2020 [34]**	■	■	■	■	■	■		■		**7**
**Gerage et al., 2015 [35]**	■	■		■	■	■				**5**
**McGregor et al., 2018 [37]**	■	■	■	■		■		■	■	**7**
**Oliveira et al., 2020 [36]**	■	■		■	■	■				**5**
**Recio-Rodriguez et al., 2013 [38]**	■	■	■	■	■	■				**6**

Each question earned one point if the answer was yes, and zero if the answer was no.

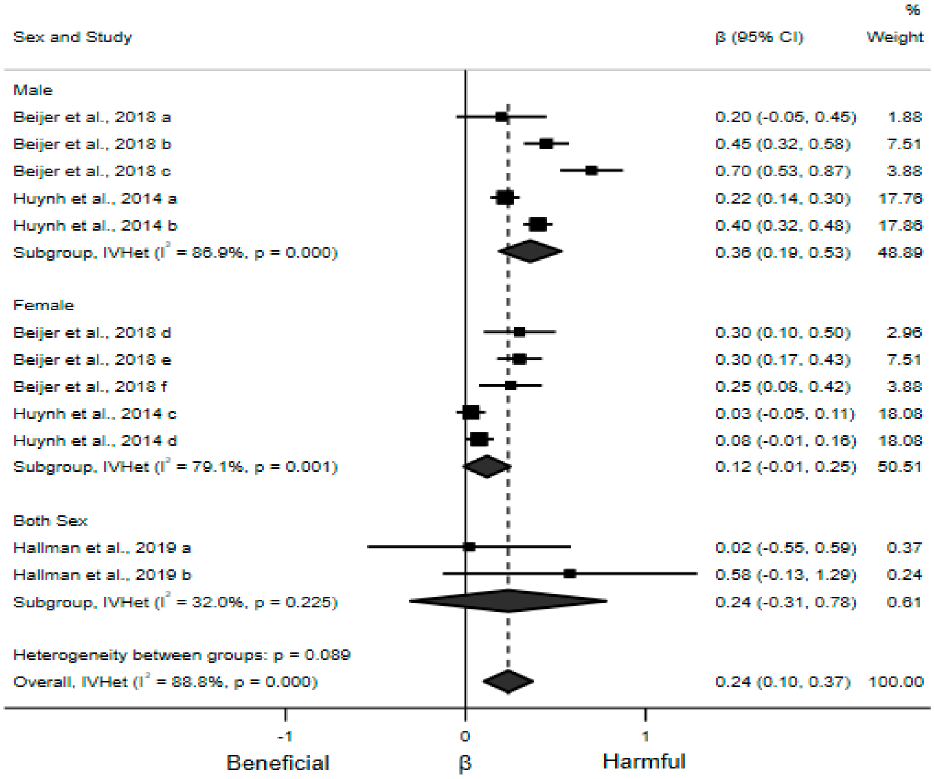

Figure S1. Forest Plots of Association between ST and HR in Subgroup Analyses by Sex.

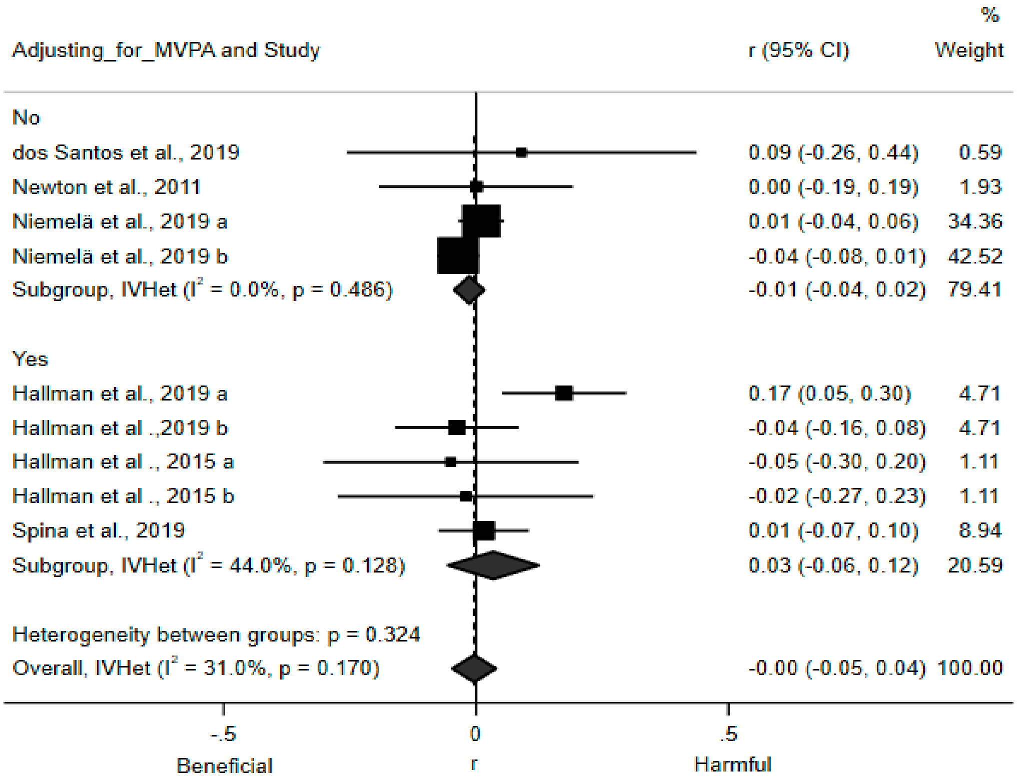
%
Adjusting_for_MVPA and Study
r (95% CI)
Weight
No
dos Santos et al., 2019
0.09 (-0.26, 0.44)
0.59
Newton et al., 2011
0.00 (-0.19, 0.19)
1.93
Niemelä et al., 2019 a
0.01 (-0.04, 0.06)
34.36
Niemelä et al., 2019 b
-0.04 (-0.08, 0.01)
42.52
Subgroup, IVHet (I^2 = 0.0%, p = 0.486)
-0.01 (-0.04, 0.02)
79.41
Yes
Hallman et al., 2019 a
0.17 (0.05, 0.30)
4.71
Hallman et al .,2019 b
-0.04 (-0.16, 0.08)
4.71
Hallman et al ., 2015 a
-0.05 (-0.30, 0.20)
1.11
Hallman et al ., 2015 b
-0.02 (-0.27, 0.23)
1.11
Spina et al., 2019
0.01 (-0.07, 0.10)
8.94
Subgroup, IVHet (I^2 = 44.0%, p = 0.128)
0.03 (-0.06, 0.12)
20.59
Heterogeneity between groups: p = 0.324
Overall, IVHet (I^2 = 31.0%, p = 0.170)
-0.00 (-0.05, 0.04)
100.00
-.5
0
.5
Beneficial
r
Harmful

(a)

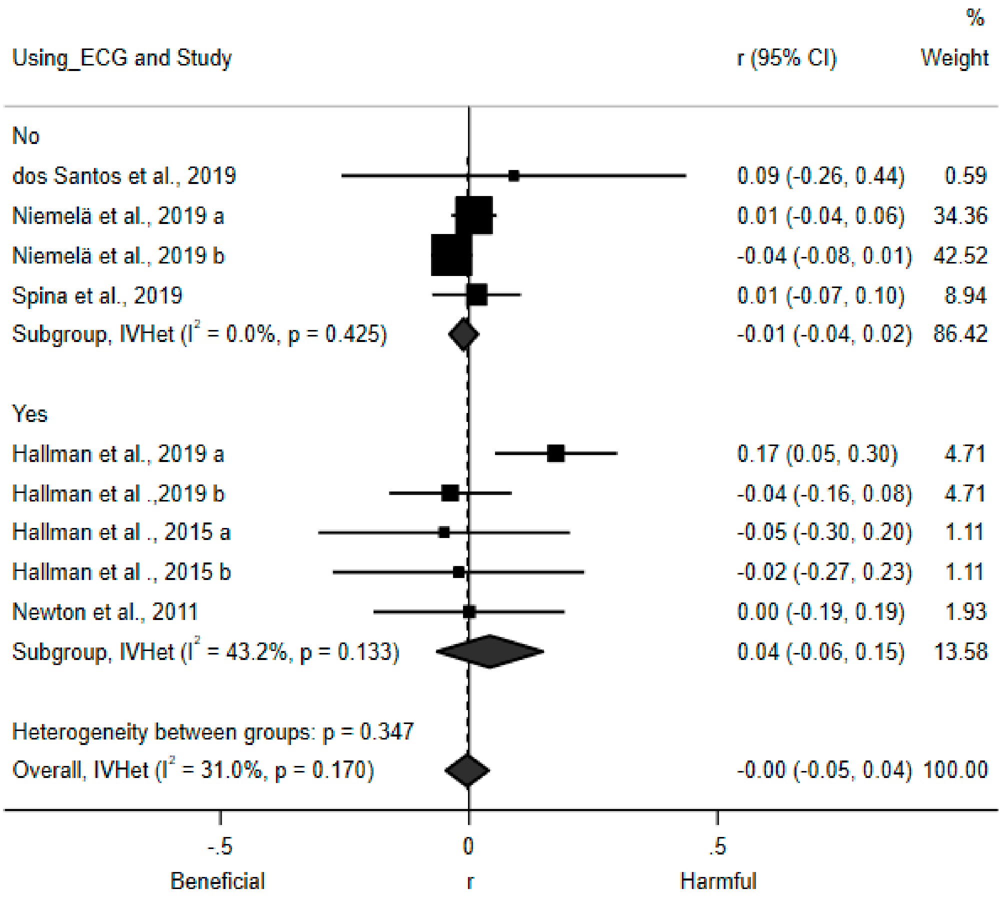

(b)

Figure S2. Forest Plots of the Association between ST and LF/HF Ratio in Subgroup Analyses.

a. Subgroup analyses by MVPA adjustment.
No indicates no adjustment for MVPA; Yes indicates adjustment for MVPA.
b. Subgroup analysis by using electrocardiogram (ECG).
No indicates not using ECG to measure HRV; Yes indicates using ECG to measure HRV.
